# Cardiovascular phenotypes in ventilated patients with COVID-19 acute respiratory distress syndrome

**DOI:** 10.1186/s13054-020-02958-8

**Published:** 2020-05-18

**Authors:** Bruno Evrard, Marine Goudelin, Noelie Montmagnon, Anne-Laure Fedou, Thomas Lafon, Philippe Vignon

**Affiliations:** 1 Medical-Surgical Intensive Care Unit, Dupuytren Teaching Hospital, 87000 Limoges, France; 2 Inserm CIC 1435, Dupuytren Teaching Hospital, 87000 Limoges, France; 3 Emergency Department, Dupuytren Teaching Hospital, 87000 Limoges, France; 4 Faculty of Medicine, University of Limoges, 87000 Limoges, France; 5 Inserm UMR 1092, Dupuytren Teaching Hospital, 87000 Limoges, France; 6 Réanimation Polyvalente, CHU Dupuytren, 2 Avenue Martin Luther king, 87042 Limoges, France

**Keywords:** Acute respiratory distress syndrome, COVID-19, Influenza, Human, Echocardiography, Echocardiography, Doppler

Approximately two-thirds of patients admitted to the intensive care unit (ICU) for coronavirus disease-19 (COVID-19) pneumonia present with the acute respiratory distress syndrome (ARDS) [1]. COVID-19-associated acute cardiac injury is frequently reported based on troponin and electrocardiographic changes [2], but its impact on cardiac function is yet unknown [3]. Accordingly, we sought to describe cardiovascular phenotypes identified using transesophageal echocardiography (TEE) in ventilated COVID-19 patients with ARDS and to compare them to those of patients with flu-induced ARDS.

All patients with confirmed COVID-19 who were mechanically ventilated for ARDS in our medical-surgical ICU underwent prospectively a TEE assessment during the first 3 days and whenever required by clinical events during ICU stay, as a standard of care. Similarly, all patients ventilated for flu-associated ARDS who underwent a TEE assessment over the last 2 years were retrospectively analyzed for comparison. Cardiovascular phenotypes were identified using previously reported TEE criteria [4]. Same applied for acute cor pulmonale (ACP) [5]. TEE studies were read by two independent experts who had no access to the cause of ARDS and examination date. Results are expressed as medians and 25th–75th percentiles. Friedman ANOVA was used to compare quantitative parameters over time in COVID-19 patients, while Mann-Whitney *U* test and Fisher’s exact test were used for comparison of continuous and categorical variables, respectively, with flu patients. No use of previous value or interpolation rule was used in the presence of missing data.

Eighteen consecutive COVID-19 patients and 23 flu patients (21 A-H1N1) were studied. COVID-19 patients were significantly older (70 [57–75] vs. 58 [49–64] years, *p* = 0.006), less severe (SAPSII 34 [30–38] vs. 43 [32–54], *p* = 0.015; SOFA 4 [2–4] vs. 6 [4–9], *p* < 0.001), required less vasopressor support (2/18 [11%] vs. 10/23 [43%], *p* = 0.038), and had longer time lag between first symptoms and ICU admission, tracheal intubation, and TEE examination when compared to flu patients (Table 1). The prevalence of left ventricular (LV) failure (3/18 [17%] vs. 14/23 [61%], *p* = 0.009), ACP (3/18 [17%] vs. 11/23 [48%], *p* = 0.051), and severe ACP (1/18 [5.5%] vs. 8/23 [35%], *p* = 0.054) was significantly lower in COVID-19 patients. Hypovolemic and hyperkinetic phenotypes were similarly observed in both groups (Table 1). Despite similar tidal volume and PEEP level, COVID-19 patients had significantly higher P/F ratio and respiratory-system compliance, and lower driving pressure than flu patients (Table 1). Pulmonary embolism was identified in none of COVID-19 patients but in one flu patient with ACP. COVID-19 patients with ACP tended to exhibit lower respiratory-system compliance (34, 32, and 30 mL/cmH_2_O) when compared to others (40 [31–45] mL/cmH_2_O). Hemodynamic profile of COVID-19 patients remained stable during the first 3 days of ICU stay (Table 2).

**Table 1:** Characteristics, presentation and outcome of ventilated patients with COVID-19 and flu-related ARDS

	COVID-19 (*n* = 18)	Flu (*n* = 23)	*p* value
Patients’ characteristics			
Age, years	70 (57–75)	58 (49–64)	0.006
Male (%)	12 (67)	12 (52)	0.524
BMI, kg/m^2^	29 (26–32)	29 (25–34)	0.519
Hypertension (%)	11 (61)	10 (43)	0.350
Diabetes mellitus (%)	4 (22)	3 (13)	0.679
Time from illness onset to ICU admission, days	11 (7–13)	5 (4–10)	0.017
Time from illness onset to intubation, days	12 (8–15)	6 (4–10)	0.002
Time from illness onset to echocardiography, days	14 (9–17)	13 (6–17)	0.001
SAPS II	34 (30–38)	43 (32–54)	0.015
SOFA score	4 (2–4)	6 (4–9)	< 0.001
Clinical presentation and treatment			
ECG changes* (%)	1 (5%)	3 (13%)	0.618
Documented coinfection (%)	3 (17)	9 (39)	0.171
Septic shock (%)	0 (%)	10 (43)	–
Vasopressor support (%)	2 (11)	10 (43)	0.038
Prone position (%)	10 (56)	14 (61)	1.000
Neuromuscular blockers (%)	17 (94%)	12 (52%)	0.005
Biology on admission			
Troponin I (ng/L)	73 (51–94)	53 (37–66)	0.020
Lactate, mmol/L	1.17 (0.89–1.57)	1.51 (1.02–2.54)	0.143
Creatinine, μmol/L	58 (42–87)	88 (59–160)	0.021
Prothombine time, %	87 (78–96)	87 (71–101)	0.979
AST, U/L	55 (27–71)	107 (46–203)	0.020
ALT, U/L	37 (27–65)	45 (27–115)	0.527
CPK, U/L	72 (34–103)	419 (180–2456)	< 0.001
White blood cell count, G/L	7.98 (6.61–11.25)	5.96 (4.02–8.05)	0.003
Lymphocyte count, G/L	0,78 (0.55–1.05)	0.75 (0.47–1.13)	0.770
Eosinophils count, G/L	0.02 (0.02–0.09)	0.01 (0.00–0.01)	0.094
Platelet count, G/L	318 (218–425)	172 (153–225)	< 0.001
Hemoglobin, g/dl	11.2 (10.2–12.3)	13.1 (11.6–14.2)	0.007
Respiratory parameters			
PaO_2_/FiO_2_	130 (81–217)	70 (62–100)	< 0.001
Arterial pH	7.35 (7.29–7.45)	7.32 (7.23–7.41)	0.121
PaCO_2_, mmHg	44 (33–51)	47 (36–60)	0.430
RR, breaths/min	24 (22–27)	25 (24–28)	0.139
Tidal volume, mL/kg	5.2 (4.5–6.2)	5.3 (4.0–6.1)	0.885
PEEP, cmH_2_O	10 (8–12)	10 (8–12)	0.476
Plateau pressure, cmH_2_O	23 (20–26)	28 (20–28)	0.144
Driving pressure, cmH_2_O	12 (10–15)	18 (17–18)	0.001
Respiratory-system compliance**, mL/cmH_2_O	38 (31–45)	23 (22–27)	0.001
Hemodynamic parameters			
Heart rate, bpm	90 (72–109)	105 (69–118)	0.494
Mean arterial blood pressure, mmHg	102 (85–110)	78 (71–94)	< 0.001
CVP, mmHg	9 (7–10)	11 (9–14)	0.058
Cardiovascular phenotypes			
ACP (%)	3 (17)	11 (48)	0.051
Severe ACP (%)	1 (5)	8 (35)	0.054
LV failure	3*** (17)	14 (61)	0.009
Hypovolemia	2 (11)	1 (4)	0.573
Hyperkinesia	6 (33)	7 (30)	1.00
Normal hemodynamic profile	8 (44)	5 (22)	0.179
Echocardiographic indices			
Cardiac index**** (L/min/m^2^)	3.1 (2.5–4.2)	2.5 (2.0–3.0)	0.034
RVEDA/LVEDA	0.55 (0.37–0.60)	0.70 (0.54–0.80)	0.021
RVFAC, %	46 (35–50)	33 (24–39)	0.002
TAPSE, mm	25 (23–29)	18 (16–22)	< 0.001
Tricuspid S′, cm/s	16.0 (15.0–20.5)	12.2 (11.0–13.4)	0.005
TR peak velocity, m/s	3.2 (2.9–3.6)	2.9 (2.4–3.2)	0.113
IVC diameter, mm	22 (19–26)	22 (21–24)	0.762
LVEF (%)	52 (44–61)	44 (28–59)	0.265
LVOT VTI, cm	22 (18–25)	18 (13–24)	0.106
Mitral E/E′ ratio	7.3 (6.5–10.9)	7.8 (6.1–10.6)	0.730
Outcome			
ICU mortality***** (%)	1 (6)	9 (39)	0.025

Abbreviations: *BMI* body mass index, *SAPSII* Simplified Acute Physiology Score, *SOFA* Sepsis Organ Failure Assessment, *AST* aspartate aminotransferase, *ALT* alanine aminotransferase, *CPK* creatinine phosphokinase, *RR* respiratory rate, *PEEP* positive end-expiratory pressure, *CVP* central venous pressure, *ACP* acute cor pulmonale, *LV* left ventricle, *RVEDA* right ventricular end-diastolic area, *LVEDA* left ventricular end-diastolic area, *RVFAC* right ventricular fractional area change, *TAPSE* tricuspid annular plane systolic excursion, *TR* tricuspid regurgitation, *IVC* inferior vena cava, *LVEF* left ventricular ejection fraction, *LVOT* left ventricular outflow tract, *VTI* velocity-time integral, *ICU* intensive care unit

*One patient had anterior negative T-wave in the COVID-19 group; 2 patients had inferior negative T-wave, and 1 patient had anterior negative T-wave in the flu group [2]

**Calculated as the tidal volume divided by the driving pressure (difference between the inspiratory plateau pressure and positive end-expiratory pressure)

***One patient was diagnosed with a Tako-tsubo syndrome during transesophageal echocardiography examination performed shortly after tracheal intubation, after 6 days of high-flow nasal cannula; full recovery of left ventricular systolic function was documented under mechanical ventilation 10 days later

****Measured using the Doppler method applied at the left ventricular outflow tract

*****As per April 24, with still 6 patients hospitalized in the intensive care unit, 5 of them being invasively ventilated

**Table 2:** Evolution of hemodynamic profile during daily transesophageal echocardiography assessments of COVID-19 patients ventilated for ARDS

	Day 1 (***n*** = 18)	Day 2 (***n*** = 10)	Day 3 (***n*** = 12)	***p*** value
Respiratory parameters				
PaO_2_/FiO_2_	130 (81–217)	128 (100–210)	137 (98–187)	0.066
PaCO_2_, mmHg	44 (33–51)	50 (32–56)	47 (37–57)	0.964
RR, breaths/min	24 (22–27)	27 (20–28)	24 (24–30)	0.651
PEEP, cmH_2_O	10 (8–12)	10 (8–13)	10 (10–12)	0.444
Plateau pressure, cmH_2_O	23 (20–26)	22 (18–27)	24 (21–27)	0.127
Driving pressure, cmH_2_O	12 (10–15)	11 (9–12)	13 (11–17)	0.368
Tidal volume, mL/kg	5.2 (4.5–6.2)	5.3 (4.6–6.6)	5.5 (4.3–6.7)	0.210
Respiratory-system compliance*, mL/cmH_2_O	38 (31–45)	33 (33–53)	37 (28–45)	0.692
Hemodynamic parameters				
Heart rate, bpm	90 (72–109)	93 (78–107)	98 (89–104)	0.368
CVP, mmHg	9 (7–10)	7 (6–10)	9 (5–13)	0.678
Mean blood pressure, mmHg	102 (85–110)	105 (87–110)	95 (84–109)	0.102
Lactate, mmol/L	1.17 (0.89–1.57)	1.85 (1.24–3.01)	1.62 (1.49–1.95)	0.264
Echocardiography indices				
Cardiac index (L/min/m^2^)**	3.1 (2.5–4.2)	2.8 (2.6–3.9)	4.1 (3.2–4.8)	0.115
RVEDA/LVEDA	0.55 (0.37–0.60)	0.53 (0.35–0.66)	0.55 (0.48–0.58)	0.549
RVFAC, %	46 (35–50)	40 (33–46)	40 (32–58)	0.821
TAPSE, mm	25 (23–29)	24 (20–28)	25 (23–28)	0.368
Tricuspid S′, cm/s	16.0 (15.0–20.5)	16.1 (14.0–18.1)	16.8 (14.9–19.9)	0.867
TR peak velocity, m/s	3.2 (2.9–3.6)	3.0 (2.7–3.7)	3.6 (2.4–3.9)	0.060
IVC diameter, mm	22 (19–26)	24 (14–30)	22 (17–24)	1.000
LVEF, %	52 (44–61)	46 (41–64)	55 (49–60)	0.549

Abbreviations: *RR* respiratory rate, *PEEP* positive end-expiratory pressure, *CVP* central venous pressure, *RVEDA* right ventricular end-diastolic area, *LVEDA* left ventricular end-diastolic area, *RVFAC* right ventricular fractional area change, *TAPSE* tricuspid annular plane systolic excursion, *TR* tricuspid regurgitation, *IVC* inferior vena cava, *LVEF* left ventricular ejection fraction

*Calculated as the tidal volume divided by the driving pressure (difference between the inspiratory plateau pressure and positive end-expiratory pressure)

**Measured using the Doppler method applied at the left ventricular outflow tract

The higher prevalence of LV failure and lower cardiac index in patients with flu-related ARDS is presumably related to septic cardiomyopathy since they sustained associated septic shock more frequently than COVID-19 patients. Depressed indices of RV systolic function and elevated central venous pressure reflecting systemic venous congestion reflect the higher prevalence of RV failure in flu ARDS patients (Table 1). This presumably results from the lower P/F, higher driving pressure, and lower respiratory-system compliance observed in this group. COVID-19 patients with ACP tended to have lower respiratory-system compliance than their counterparts, presumably due to distinct ARDS phenotypes [6]. This pilot study is limited by its small sample size and the retrospective comparison with historical flu-related ARDS patients.

This first study assessing hemodynamically ventilated COVID-19 patients with TEE shows a lower prevalence of LV and RV failure than in flu-related ARDS patients. Whether herein reported cardiovascular phenotypes are influenced by the type of COVID-19 ARDS remains to be determined [6]. These preliminary data warrant confirmation in large-scale multicenter cohorts.

## Data Availability

N/A

## Abbreviations

ACP: Acute cor pulmonale
ARDS: Acute respiratory distress syndrome
COVID-19: Coronavirus disease 2019
ICU: Intensive care unit
LV: Left ventricle
RV: Right ventricle
SAPS II: Simplified acute physiology score II
SOFA: Sepsis-related organ failure assessment
